# Severity of Financial Toxicity for Patients Receiving Palliative Radiation Therapy

**DOI:** 10.1177/10499091231187999

**Published:** 2023-07-05

**Authors:** Jeremy P. Harris, Eric Ku, Garrett Harada, Sophie Hsu, Elaine Chiao, Pranathi Rao, Erin Healy, Misako Nagasaka, Jessica Humphreys, Michael A. Hoyt

**Affiliations:** 1 Department of Radiation Oncology, 8788 University of California Irvine, Orange, CA, USA; 2 Department of Medicine, Division of Hematology/Oncology, 8788 University of California Irvine, Orange, CA, USA; 3 Department of Geriatrics and Extended Care, Division of Palliative Care, 19974 Tibor Rubin VA Medical Center, Long Beach, CA, USA; 4 Department of Medicine, Division of Palliative Medicine, 8785 University of California, San Francisco, CA, USA; 5 Department of Population Health & Disease Prevention, 8788 University of California Irvine, Irvine, CA, USA

**Keywords:** FACIT-COST, financial toxicity, health-related quality of life, metastatic cancer, palliative radiation, radiation therapy

## Abstract

**Introduction:** Financial toxicity has negative implications for patient well-being and health outcomes. There is a gap in understanding financial toxicity for patients undergoing palliative radiotherapy (RT). **Methods:** A review of patients treated with palliative RT was conducted from January 2021 to December 2022. The FACIT-COST (COST) was measured (higher scores implying better financial well-being). Financial toxicity was graded according to previously suggested cutoffs: Grade 0 (score ≥26), Grade 1 (14-25), Grade 2 (1-13), and Grade 3 (0). FACIT-TS-G was used for treatment satisfaction, and EORTC QLQ-C30 was assessed for global health status and functional scales. **Results:** 53 patients were identified. Median COST was 25 (range 0-44), 49% had Grade 0 financial toxicity, 32% Grade 1, 15% Grade 2, and 4% Grade 3. Overall, cancer caused financial hardship among 45%. Higher COST was weakly associated with higher global health status/Quality of Life (QoL), physical functioning, role functioning, and cognitive functioning; moderately associated with higher social functioning; and strongly associated with improved emotional functioning. Higher income or Medicare or private coverage (rather than Medicaid) was associated with less financial toxicity, whereas an underrepresented minority background or a non-English language preference was associated with greater financial toxicity. A multivariate model found that higher area income (HR .80, *P* = .007) and higher cognitive functioning (HR .96, *P* = .01) were significantly associated with financial toxicity. **Conclusions:** Financial toxicity was seen in approximately half of patients receiving palliative RT. The highest risk groups were those with lower income and lower cognitive functioning. This study supports the measurement of financial toxicity by clinicians.

## Introduction

Palliative radiotherapy (RT) is routinely indicated for solid tumors that have become symptomatic. Tumors warranting palliation can occur anywhere in the body, with common areas being bones, brain, spine, and chest.^1,2^ It is estimated that palliative RT is employed for 9%-39% of patients with metastatic cancer in the US.^3–6^ Treatment of the patient necessarily entails consideration of the burden of treatment-related costs, which is known as financial toxicity.^7^ Financial burden can extend from out-of-pocket expenses, time away from work, travel and parking fees, and caregiving needs, and it is likely to be linked to health-related quality of life (HRQoL), patient well-being, and possibly patient mortality.

Palliative RT treatments are generally provided to those with incurable disease, often at the end of life when patients are physically and financially suffering.^3^ Understanding the patient-reported experience is now critical for drug development assessment by the Food and Drug Administration (FDA) and a major interest of the NCI.^8,9^ Efforts have focused on HRQoL and treatment toxicity reports.^10^

Little is known about patients’ experience of financial toxicity and its impact on HRQoL with palliative RT. The gap in understanding financial toxicity represents an important unmet need, since economic loss and bankruptcy are common among patients with metastatic cancer.^11^ Despite a declining incidence of many cancers, due to an aging population, longer survival after diagnosis, and more expensive therapies, the national cost of care for cancer patients is projected to grow 30% from 2015 to 2030.^12^

The goal of this study was to determine the degree of financial toxicity in a population undergoing palliative RT, as well as identify relationships between financial toxicity and HRQoL in this patient group.

## Methods

### Patients and Procedures

A review of patients referred for palliative RT at an NCI-designated Comprehensive Cancer Center was conducted from January 2021 to December 2022. Patients were included if they were treated with palliative intent RT for any cancer diagnosis and prior to treatment had completed a financial toxicity survey that is routinely administered in the course of clinical care. This was a retrospective study approved by the institutional review board.

### Measures

Financial toxicity was determined with the FACIT-COST (COST), a validated 12-item self-report measure of financial well-being yielding scores between 0 and 44 (Table 1).^13^ Higher scores imply better financial well-being. Financial toxicity scores were graded according to previously suggested cutoffs for the COST: Grade 0 (score ≥26), Grade 1 (14-25), Grade 2 (1-13), and Grade 3 (0).^14,15^

**Table 1.** FACIT-COST v2 Survey Items. Respondents Were Asked to Indicate a Response as it Applied to the Past 7 Days. For Each Question, the Allowed Responses Were ‘not at all’, ‘a Little’, ‘Somewhat’, ‘Quite a Bit’, and ‘Very Much’. Copyright 2014, FACIT and The University of Chicago.

FT1. I know that I have enough money in savings, retirement, or assets to cover the costs of my treatment
FT2. My out-of-pocket medical expenses are more than I thought they would be
FT3. I worry about the financial problems I will have in the future as a result of my illness or treatment
FT4. I feel I have no choice about the amount of money I spend on care
FT5. I am frustrated that I cannot work or contribute as much as I usually do
FT6. I am satisfied with my current financial situation
FT7. I am able to meet my monthly expenses
FT8. I feel financially stressed
FT9. I am concerned about keeping my job and income, including work at home
FT10. My cancer or treatment has reduced my satisfaction with my present financial situation
FT11. I feel in control of my financial situation
FT12. My illness has been a financial hardship to my family and me

In addition, the 8-item FACIT-TS-G (Version 4) was reviewed from the first follow-up appointment to assess treatment satisfaction. To determine HRQoL, the EORTC QLQ-C30, a 30-item patient-reported outcome measure that consists of a score of global health status and functional scales, which include physical functioning, role functioning, emotional functioning, cognitive functioning, and social functioning domains (Version 3.0).^16,17^ Higher scores for the functioning scales and global health status denote better functioning.

Demographic, clinical, and treatment variables were extracted from the electronic medical record. Hospital admissions within the prior 12 months were determined. Socioeconomic status was further characterized by area income, defined as the median family income in the patient’s census tract using 2020 Census Data from the Federal Financial Institutions Examination Council.^18^

### Data Analytic Approach

Summary scores for the COST and FACIT-TS-G and scores from the EORTC global health status and functional scales were computed.^19^

Multiple imputations by chained equations using predictive mean matching were computed and used for incomplete survey responses. Spearman’s rank correlation coefficient, Kruskal-Wallis testing, and linear regressions were conducted to determine associations between COST and demographic, clinical, and patient-reported outcome (PRO) variables.

Multivariate logistic regression was done for the presence of financial toxicity (ie, COST <26), with the initial model incorporating any variable significant (*P* < .1) from univariate modeling. The final model was determined from backward stepwise selection using Akaike Information Criterion (AIC) corresponding to a relative likelihood of 5% and confirmed with likelihood ratio testing. Internal validation was done by generating 100 bootstrap samples with replacement, and model performance was assessed with area under the curve (c-index). Optimism-corrected performance was calculated by subtracting the mean difference between bootstrap and test performance from the c-index.

The Kaplan-Meier method was used to estimate survival from the time of survey, and Cox proportional hazards modeling was used to measure associations with overall survival (OS).

## Results

In total, 53 patients were identified who had completed PRO surveys. Median age was 62 years, 51% were female, 54% white race, and 89% spoke English as their preferred language (Table 2). Insurance coverage included 49% commercial payers, 30% Medicare, and 21% Medicaid. The median area income was $98,958 (range $32,303-$190,833), and median distance to the treatment center was 11.6 miles (range 2.4-78.6 miles).

**Table 2.** Clinical and Demographic Characteristics of Study Population.

		n	% or Range
Race and ethnicity			
	White	29	55%
	Underrepresented minority	13	25%
	Asian	11	21%
Preferred language			
	English	47	89%
	Spanish	3	6%
	Korean	1	2%
	Vietnamese	2	4%
Insurance			
	Medicare	16	30%
	Medicaid	11	21%
	Commercial payer	26	49%
Area income	Median (range, $)	$98,958	($32,303-$190,833)
Distance to treatment center	Median (range, miles)	11.6	(2.4-78.6)
Performance status			
	0	4	8%
	1	40	75%
	2	9	17%
BMI	Median (range)	24.6	(18.5-43.4)
Primary malignancy			
	Lung	25	47%
	Cutaneous	10	19%
	Colorectal	4	8%
	Breast	3	6%
	Bone	3	6%
	Sarcoma	2	4%
	Thymic	2	4%
	Renal	1	2%
	Esophageal	1	2%
	Lymphoma	1	2%
	Thyroid	1	2%
Time from diagnosis	Median (range, months)	13	(0-106.0)
Number of prior chemotherapy regimens			
	0-1	33	62%
	≥2	20	38%
Hospitalizations			
	0-1	45	85%
	≥2	8	15%
Irradiated site			
	Bone (spine)	12	23%
	Brain	10	19%
	Bone (non-spine)	9	17%
	Lung/mediastinum	9	17%
	Skin	6	11%
	Abdomen	3	6%
	Neck	2	4%
	Pelvis	2	4%
Radiation dose	Median (range, Gy)	25	(4.0-45.0)
Radiation dose, BED	Median (range, Gy)	39	(4.8-81.6)
Radiation fractions			
	1-5	32	60%
	≥6	21	40%
Radiation technique			
	3D	37	70%
	IMRT	6	11%
	SRS	10	19%

BED, Biologically effective dose presented with α/β = 10 Gy; IMRT, Intensity modulated radiation therapy; SRS, stereotactic radiosurgery.

Time from cancer diagnosis was a median of 13 months (range 0-106 months), and 38% of patients had received ≥2 prior systemic therapies. The most common irradiated sites included spine (23%), brain (19%), non-spine bones (17%), and lung/mediastinum (17%). The median RT dose was 25 Gy (range 4-45 Gy), corresponding to a median BED_α/β = 10Gy_ of 39 Gy (range 4.8-81.6 Gy). 70% of patients were treated with 3D technique, 11% with intensity modulated radiation therapy, and 19% with stereotactic radiosurgery.

**Figure 1.**
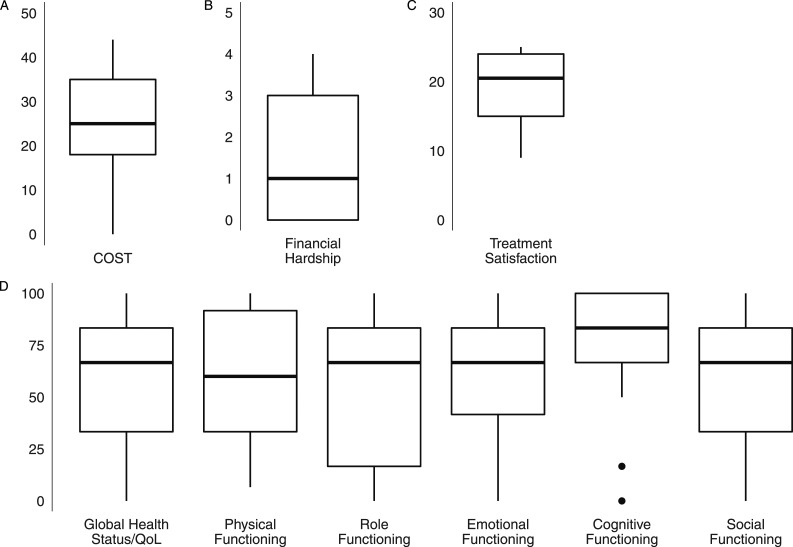
Distribution of (A) COST, (B) Financial Hardship (COST question 12), (C) treatment satisfaction (FACIT-TS-G), and (D) EORTC global health status/QoL and functional scales. Lower COST indicates higher financial toxicity, higher financial hardship indicates higher toxicity, higher treatment satisfaction and EORTC functional scales indicate improved quality of life.

Median COST was 25 (range 0-44), with lower scores indicating greater financial toxicity (Figure 1). Nearly half of the sample (49%) reported grade 0 financial toxicity, 32% had grade 1 financial toxicity, 15% had grade 2 financial toxicity, and 4% had grade 3 financial toxicity. Overall, cancer caused financial hardship among 45% (patient-reported COST question 12).

Lower COST (ie, more financial toxicity) was weakly associated with global health status/QoL (rho = −.28, *P* = .04), physical functioning (rho = −.35, *P* = .01), role functioning (rho = −.32, *P* = .02), and cognitive functioning (rho = −.34, *P* = .01); moderately associated with social functioning (rho = −.44, *P* = .001); and strongly associated with emotional functioning (rho = −.61, *P* < .0001) (Table 3). Having a lower COST was also associated with higher financial hardship from question 12 of COST (rho = .87, *P* < .0001).

**Table 3.** Associations Between a Lower COST (higher financial toxicity) and Clinical Variables. Lower COST (Positive Rho or Positive Beta) Correspond to More Financial Toxicity. Non-Parametric Models were Included Spearman’s Rank Correlations and Kruskal-Wallis Tests. Univariate Linear Regressions were used for Parametric Modeling. Multivariate Logistic Regression was for the Presence of Financial Toxicity (Grade 1-3), with the Initial Model Incorporating any Variable Significant (*P* < .1) From Univariate Modeling. The Final Model was Determined From Backward Stepwise Selection Using AIC.

		Non-parametric Models		Univariate Linear Regression Models		MVA Logistic Regression Initial		MVA Logistic Regression Final	
		Rho	P	Beta	P	OR	P	OR	P
Age (per 10 years)		−.17	.23	−1.58	.23				
Sex (female vs male)		−.006	.97	−.10	.98				
Race and ethnicity			.005						
	White			Ref					
	Underrepresented minority			12.74	.001	8.77	.10		
	Asian			8.64	.03	.39	.48		
Marital status			.83						
	Married			Ref					
	Single			1.12	.80				
	Divorced			4.30	.47				
	Widowed			4.05	.54				
Primary language is English		−.39	.004	−15.82	.002	3.76E-09	.99		
Insurance type			.01						
	Medicare			−13.74	.003	1.41	.84		
	Medicaid			Ref					
	Commercial payer			−13.40	.001	2.19	.58		
Area income (per $10,000)		−.45	.0006	−1.24	.0006	.77	.06	.80	.007
Distance to treatment center (per 10 miles)		−.06	.64	−.67	.53				
Performance status			.91						
	0			−2.90	.66				
	1			Ref					
	≥2			1.24	.79				
BMI		−.17	.24	.39	.19				
Number of prior chemotherapy regimens (≥2 vs 0-1)		.13	.35	−4.08	.24				
Number of hospitalizations (≥2 vs 0-1)		`	.28	−4.44	.35				
Radiation dose, BED (per 10 Gy)		−.04	.75	.14	.90				
Radiation fractions (≥6 vs 1-5)		−.11	.44	−3.43	.52				
Radiation technique			.88						
	3D			Ref					
	IMRT			2.05	.71				
	SRS			−2.28	.61				
Date of survey (per month)		−.06	.65	−.16	.67				
Treatment satisfaction		−.19	.35	−.39	.41				
Global health status/QoL		−.28	.046	−.12	.05	1.06	.04		
Physical functioning		−.35	.01	−.13	.02	.98	.21		
Role functioning		−.32	.02	−.10	.03	.99	.70		
Emotional functioning		−.61	<.0001	−.26	<.0001	.98	.38		
Cognitive functioning		−.34	.01	−.11	.08	.94	.03	.96	.01
Social functioning		−.44	.001	−.16	.002	.98	.30		
Financial hardship		.87	<.0001	6.50	<.0001				

**Figure 2.**
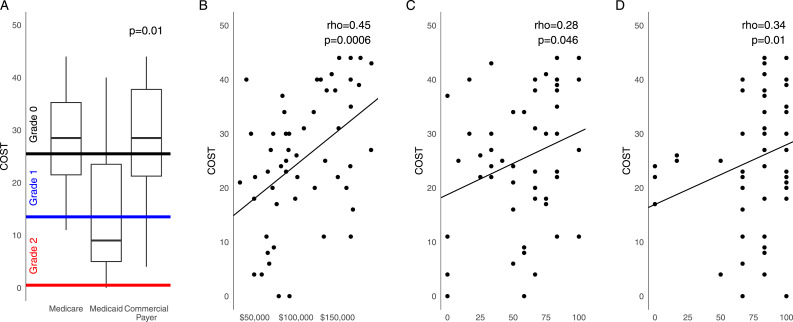
Financial toxicity according to (A) insurance type, (B) area income, (C) global health status/QoL, and (D) cognitive functioning. Grade 0 financial toxicity corresponded to COST ≥26, Grade 1 to COST 14-25, Grade 2 to COST 1-13, and Grade 3 to COST = 0.

Examination of demographic factors revealed that higher income was inversely associated with financial toxicity (rho = −.45, *P* = .0006) (Figure 2). Having Medicare (*P* = .003) or private (*P* = .001) coverage (rather than Medicaid) was associated with less financial toxicity, whereas having an underrepresented minority background (*P* = .001) or having a non-English language preference (rho = .39, *P* = .003) was associated with greater financial toxicity.

Multivariable logistic regression for the presence of financial toxicity was done (Table 3). The final model after backward selection on AIC included higher area income (HR .80, *P* = .007), and higher cognitive functioning (HR .96, *P* = .01). Likelihood ratio testing was performed (*P* = .17, indicating goodness of fit). Internal validation was done with bootstrap sampling, with an initial c-index of .813, and optimism corrected c-index of .796.

Median follow up was 8.1 months, and median 6-month survival was 85% (95% CI 75%-96%). Inferior OS was associated with having ≥2 prior systemic therapies (HR 3.17, *P* = .04), but not with COST (HR 1.01, *P* = .69). Early death within the first 3 or 6 months was not strongly associated with financial toxicity (rho = −.043, *P* = .71, and rho = −.071, *P* = .78 respectively).

## Discussion

Financial toxicity and financial hardship were seen in approximately half of patients receiving palliative RT (51% and 45%, respectively). Patient-reported global health status and functional domains correlated with financial toxicity. On multivariate analysis, area income and cognitive functioning were independently associated with financial toxicity.

In the initial validation studies of the COST measure, a model was trained on 233 patients with stage IV cancer receiving chemotherapy, and validated on 367 patients with a diagnosis of thyroid cancer.^13^ Financial toxicity was seen in 58% of patients in both the training and validation sets, and it explained 7.6% of the variability in HRQoL. There are no known studies using the COST measure specific to the population receiving palliative RT; however, in 1 study of 63 patients who received RT for head and neck cancer, the median COST was 26.5.^20^ Similar to that of current study, financial toxicity was associated with area income, and those at higher risk were also more likely to miss clinic visits, require infusions, and require a feeding tube. In a researcher-designed survey of 157 patients, RT was associated with certain aspects of financial toxicity, including time away from one’s job, loss of income, and additional costs with transportation.^21^ Such results highlight the financial vulnerabilities of RT patients, as radiation treatment requires daily sessions over days or weeks, which poses a risk to maintaining income unique from other cancer treatments. Interestingly, cognitive functioning remained significantly associated with financial toxicity in multivariate analysis. The cognitive functioning domain pertains to concentration and memory, and the potential predictive value for financial toxicity requires further validation.

Nationally, the cost of cancer care for the last 12 months of life for patients makes up about 28% of costs overall.^12^ Cost-conscious approaches to care and financial services are particularly relevant for this group. Since there is tremendous overlap between the therapeutic goals of palliative RT and services from palliative care specialists, it is ripe for organizational integration.^22^ For example, the multidisciplinary clinic model has shown success in shortening radiation schedules and boosting the portion who receive palliative care at the end-of-life.^23^ However, in 1 study less than half of patients treated with palliative RT were also receiving palliative care.^24^

Although financial toxicity is understudied in the population receiving RT, physicians are taking note of the importance of this issue. In a survey of 210 radiation oncologists, 53% were “very concerned” with treatment-related costs negatively affecting their patients.^21^ This may represent a missed opportunity, since there tends to be a disconnect between the patient and physician experience of cost discussions.^25^ But when physicians engage in communication around costs, it can result in a decrease in expenditures and improvement in medical advice adherence.^26,27^ One effective step to meet this need will involve education in cost discussions across levels of practice and training.^28^

Reduced access to in-person care related to the COVID-19 pandemic has exacerbated financial burden for some patients. In 1 cross-sectional study, those participating in telehealth visits were significantly more likely to be worried about their future financial problems.^29^ In an online survey study, anxiety about COVID-19 correlated with higher levels of financial toxicity.^30^ In the current study, there was no observed association between the date of the survey and COST scores. However, the entire study occurred after the beginning of the pandemic. Additionally, it is not clear how financial strain and toxicity has changed as the course of the pandemic has evolved. Serial measurements will be needed to better assess the impact of COVID-19 and provide a more accurate picture of trends over time.^31^

The findings of the current study are supported by similar reports that lower income and lower income area are risk factors for financial toxicity across many cancers, including breast, colorectal, lung, prostate, gynecologic, and hematologic malignancies.^32–37^ This study reproduces findings in other research demonstrating that those from racial and ethnic minority groups are at higher risk for financial toxicity.^32,38^ Thus, the current study highlights the importance of measuring financial toxicity in these high-risk groups.

Cancer treatments are expensive to patients, as noted by a survey of patients who did not receive any financial assistance, in which average out-of-pocket expenditures were $708 per month.^39^ For patients who utilized copayment assistance programs, expenditures were lower, but they were more likely to reduce spending on basics like food and clothing (51%), borrow money or used credit to pay for medications (42%), and partially fill prescriptions (24%). Patients with multiple physical or mental health comorbidities also have more healthcare-associated costs and out-of-pocket expenditures.^40,41^ As a result, long-term survivors of cancer risk being faced with large medical bills, with 1 survey of 4517 patients finding 34% suffered debt due to their cancer.^11^ For those who file bankruptcy, healthcare outcomes and mortality are higher.^42^

Approximately half of patients in the current study had government sponsored insurance (Medicare, Medicaid). While out-of-pocket expenditures vary widely within the US, the high risk of financial toxicity with cancer patients is international, including for countries with universal or individual payer systems.^43–47^ In fact, financial toxicity is significantly greater outside the US, in countries with lower average incomes.^48^ While COST is widely-used, and a psychometrically validated measure of financial toxicity, its design and use maybe specific to the US healthcare context.^31^ Work is needed to be able to extend this measurement tool to other populations.^49^

One of the strengths of this study is the socioeconomic diversity of the sample. However, a notable limitation is that the survey was administered solely in English. Additionally, all patients seen in the radiation oncology clinic had some form of health insurance, including Medicaid given on an emergency basis for those hospitalized with a new cancer diagnosis. The population of uninsured and non-English speaking patients likely represent a group at even higher risk for financial toxicity, and generalizability of the current study to those populations is not advised. In addition, the suggested grading system and validation of financial toxicity utilizing COST was done with the FACT-G and presented in abstract form.^15^ While convenient, the grading system has generally not used clinically. Other limitations include selection bias, response bias, and measurement bias due to the single-institutional design, the fact that the COST survey was designed from a sample of insured participants in the US, and that area income may not adequately describe the income for the studied population. Lastly, the sample size was limited by inclusion of patients at a single cancer center, and model overfitting was possible despite the attempts at internal validation.

## Conclusions

In conclusion, financial toxicity and financial hardship are prevalent in the population receiving palliative radiation. The highest risk groups are those with lower income and lower cognitive functioning. This study supports the hypothesis that financial toxicity is a common and unique adversity that should be measured in clinics seeing cancer patients.
